# Development of a comprehensive list of criteria for evaluating consumer education materials on colorectal cancer screening

**DOI:** 10.1186/1471-2458-13-843

**Published:** 2013-09-13

**Authors:** Maren Dreier, Birgit Borutta, Gabriele Seidel, Inga Kreusel, Jürgen Töppich, Eva M Bitzer, Marie-Luise Dierks, Ulla Walter

**Affiliations:** 1 Institute for Epidemiology, Social Medicine and Health Systems Research, Hannover Medical School, Carl-Neuberg Str. 1, 30625 Hannover, Germany; 2 Department 2, Federal Centre for Health Education (BZgA), Cologne, Germany; 3 Department of Public Health and Health Education, University of Education, Freiburg, Germany

**Keywords:** Screening, Early diagnosis, Colorectal cancer, Bowel cancer, Informed decision-making, Informed choice, Evidence-based health information, Evidence-based patient information, Risk communication, Decision aids

## Abstract

**Background:**

Appropriate patient information materials may support the consumer’s decision to attend or not to attend colorectal cancer (CRC) screening tests (fecal occult blood test and screening colonoscopy). The aim of this study was to develop a list of criteria to assess whether written health information materials on CRC screening provide balanced, unbiased, quantified, understandable, and evidence-based health information (EBHI) about CRC and CRC screening.

**Methods:**

The list of criteria was developed based on recommendations and assessment tools for health information in the following steps: (1) Systematic literature search in 13 electronic databases (search period: 2000–2010) and completed by an Internet search (2) Extraction of identified criteria (3) Grouping of criteria into categories and domains (4) Compilation of a manual of adequate answers derived from systematic reviews and S3 guidelines (5) Review by external experts (6) Modification (7) Final discussion with external experts.

**Results:**

Thirty-one publications on health information tools and recommendations were identified. The final list of criteria includes a total of 230 single criteria in three generic domains (formal issues, presentation and understandability, and neutrality and balance) and one CRC-specific domain. A multi-dimensional rating approach was used whenever appropriate (e.g., rating for the presence, correctness, presentation and level of evidence of information). Free text input was allowed to ensure the transparency of assessment. The answer manual proved to be essential to the rating process. Quantitative analyses can be made depending on the level and dimensions of criteria.

**Conclusions:**

This comprehensive list of criteria clearly has a wider range of evaluation than previous assessment tools. It is not intended as a final quality assessment tool, but as a first step toward thorough evaluation of specific information materials for their adherence to EBHI requirements. This criteria list may also be used to revise leaflets and to develop evidence-based health information on CRC screening. After adjustment for different procedure-specific criteria, the list of criteria can also be applied to other cancer screening procedures.

## Background

Since the 70s, cancer screening procedures have gained increasing significance for public health and are promoted in many countries. However, in recent years, it became clear that the communication of some procedures may have overemphasized their benefits and disregarded their risks [1]. Potential harms of cancer screening include adverse effects from the procedure itself, overdiagnosis, and false-positive results, including the mental stress and/or unnecessary diagnostic tests resulting there from [2]. For this reason and for ethical reasons that apply to all medical procedures with potential side effects, participants must give their informed consent before screening [3,4]. In Germany, persons aged 50 and older who have statutory health insurance have free access to colorectal cancer (CRC) screening tests, including the fecal occult blood test (FOBT) and (since 2002) screening colonoscopy. As colonoscopy is an invasive and burdensome procedure with potentially lethal complications (very rare) [5,6], special efforts are needed to ensure informed decision-making [3].

Like other health communication strategies, written health information materials support informed choices regarding whether or not to attend CRC screening and have certain tests performed [7,8]. Appropriate information should meet evidence-based health information (EBHI) standards [9]. Accordingly, it must include balanced, unbiased, quantified, understandable, and evidence-based information about CRC and the potential benefits and harms of the screening procedures [10]. Numerous tools to evaluate the quality of health-related information are available. However, we found no tool or checklist that systematically evaluates health information on cancer screening procedures according to EBHI standards. The existing tools focus on criteria for the characterization of structural and process quality (e.g., DISCERN [11]), self-assessment of lay persons (e.g., Check-In [12]), or a reviewer’s subjective judgment of specific benefits and harms [13]. Collected information about structural and process quality is typically used as a surrogate marker for parameters of outcome quality. For example, editorial independence stands for balanced information or clarity, and layout for understandability. None of the generic tools, including the International Patient Decision Aid Standards Instrument (IPDASi) [14], directly evaluates reliability and understandability – two important features of EBHI. Thus, false or biased information may be rated as appropriate just because formal standards are met.

In Germany, many different players in healthcare provide information about CRC screening. These include governmental organizations, foundations, healthcare providers, and health insurance companies. Presumably, not all of the existing information meets EBHI standards, but rather depicts only the benefits without harms and/or strongly encourages participation in screening [15]. The German National Cancer Plan [16] was initiated by the Federal Ministry of Health in 2008 to develop and improve cancer screening and care of cancer patients. One aim is to enhance consumer information materials on the benefits and risks of screening procedures to support informed decision-making regarding whether to attend screening or not. In this context, the Federal Ministry of Health initiated a project on CRC screening to identify consumer education materials in conformity with EBHI standards. Initially, we developed a list of criteria that helps experts systematically assess whether the available flyers and brochures provide reliable, correct, understandable and unbiased information on CRC screening. The underlying concepts and methods of the development process as well as the resulting list of criteria and its strengths and weaknesses are presented in this article. Rating examples are provided to illustrate the application of this instrument.

## Methods

### Study design

The whole research project included steps 1) to identify consumer education materials on CRC 2) to develop an assessment tool for experts’ use 3) to assess the identified materials from experts’ view, and 4) to assess the materials from consumers’ view. The study protocol was approved by the ethics committee of Hannover Medical School (Application No. 1803–2013). In this article we focus on the development of the assessment tool.

**Figure 1:**
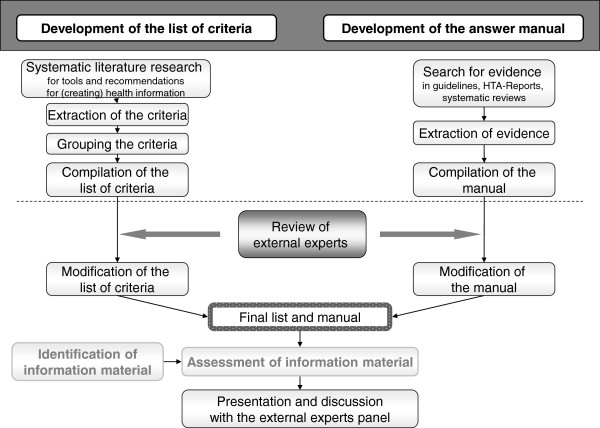
Study design used to develop the list of criteria and answer manual.

The main goal was to produce a comprehensive list of criteria based on EBHI requirements for detailed assessment of the contents and correctness of health information on CRC screening and to make such assessments as objective as possible. Criteria were extracted from recommendations on EBHI and supplemented with criteria from previous health information assessment tools. The extracted criteria were sorted and categories and subcategories were defined. The list of criteria was developed in the following steps (Figure 1):

(1) Systematic literature search to identify recommendations and assessment tools for health information

(2) Extraction of the identified criteria

(3) Grouping of the criteria into categories and a comprehensive list of criteria

(4) Review of the list of criteria by external experts

(5) Modification of the list of criteria

(6) Creation of an answer manual

(7) Discussion with external experts

These steps are described in detail in the following sections. As shown in Figure 1, we also rated German flyers and brochures on CRC screening with the developed list of criteria. The results are not shown, but rating examples from that part of the project will be used to illustrate how to apply the instrument.

### Methodological considerations

The derived a priori methodological considerations for the list of criteria were as follows:

(1) Criteria will not ask for aggregated information, if possible. For example, instead of asking for “any” adverse effects, it will ask in detail about single adverse effects like bleeding, pain, and perforation in order to prevent the reviewer from assessing combined information.

(2) If possible, there will be no multi-level response options (e.g., Likert items), but rather “yes”, “no” or “unclear”. *Rationale*: The goal is not to obtain levels of agreement or disagreement, as with psychometric tools, but unambiguous statements.

(3) The direction of the response options shall be adjusted in such a way that “yes” always corresponds to a rating of “adequate” or “appropriate”.

(4) No numerical rating or sum score will be used (no use of scales yielding a summary score). *Rationale*: Summary scores imply an implicit weighting of the criteria that is not evidence-based but arbitrary.

(5) Except for formal issues (specification of authors, publication date, etc.), each reviewer shall not only document whether specific information was reported but also whether it was correct, (e.g., whether the risk of bleeding was reported, and whether it was reported *correctly*).

(6) Reporting about evidence levels or non-sufficient evidence will be recorded.

(7) Each rating will be accompanied by the corresponding quotation from the information source to ensure the transparency of assessment.

(8) A detailed, evidence-based answer manual will be developed and implemented in order to achieve consistent ratings.

(9) Assessment of the information material will be carried out by two independent reviewers, and any discrepancies will be resolved by consensus with a third reviewer.

### Systematic literature search

A comprehensive systematic literature search was carried out in 13 electronic databases, including EMBASE and Medline. The search included health information recommendations and assessment tools for information on the underlying disease. It was restricted to articles in English or German published from 1/2000 to 8/2010. The search strategy involved the following keyword combinations and their German translations: “criteria”, “quality”, “quality criteria”, “checklist”, “evaluation” or “assessment” combined with the terms ”decision support for patient-informed decision-making”, “patient information”, “shared decision/decision making”, “risk communication”, “health information”, “evidence-based patient information” and “information brochures”. This search yield a total of 3,097 documents that were stepwise selected on the level of title, abstract and full text. A manual search was carried out based on the references in the identified publications. Additionally, a web-based search was performed with the Google search engine using combinations of keywords similar to those used in the database research. The first 50 results of each of the 19 search terms were evaluated for appropriateness. The detailed strategy used for the database search and the web-based search is listed in the Appendix (see Additional file 1). Two independent reviewers screened and selected the articles.

### Extraction and categorization of criteria

From the identified documents, two researchers (MD, BB) extracted and grouped criteria on formal issues, CRC, CRC screening procedures, neutrality and balance, while two others (GS, IK) extracted criteria on presentation and understandability. The resulting list of criteria was approved by the whole project team. These researchers also applied the list of criteria in step 3 of this project that is not within the scope of this article.

### Answer manual

A manual providing the correct answers for each criterion was developed. The aim was to minimize the subjectivity of ratings and achieve clear and unambiguous assessments. Whenever possible, the correct answers were derived from selective literature searches focusing on evidence from systematic reviews, HTA reports or S3 guidelines. Evidence levels were assigned according to the Oxford Centre of Evidence-based Medicine [17].

### Review by external experts

Twelve external experts in the field of (colorectal) cancer screening, including patient representatives and staff from governmental health-related institutions, cancer research institutes, providers of healthcare services, and statutory health insurances were asked to review the preliminary list of criteria. Six experts responded, providing feedback in a telephone interview with two researchers. Their comments were recorded instantly. Modifications proposed by the experts were discussed within the project team and, if approved, implemented into the list of criteria. The final list of criteria was discussed in a meeting with the experts.

## Results

### Development of the list of criteria

Fifteen documents with recommendations and 16 with assessment tools for health information were identified. Among the recommendations, n = 2 referred to cancer screening [16,18], n = 1 to screening [19], and n = 1 to orthopedic interventions [20]; n = 11 had no special focus [4,9,21-29]. Among the assessment tools, n = 1 referred to colorectal cancer screening [15], n = 1 to diagnostic breast tests [30], n = 3 refer to mammography screening [31-33], and n = 2 to patient decision aids [14,34], and n = 7 had no special focus [11,12,35-41]. Criteria for assessing health information were systematically extracted from these documents, and the single criteria were grouped into seven categories (Table 1): formal issues, information on CRC screening, information on screening colonoscopy, information on the fecal occult blood test, readability/comprehensibility, layout and neutrality and balance. These categories were further aggregated into four domains, one representing CRC-specific content issues and three describing generic issues applicable to different cancer screening procedures.

**Table 1:** Content structure of the list of criteria for evaluating consumer information materials on colorectal cancer (CRC) screening (n = 230 criteria*)

	**Domain (n criteria)**	**Category (n criteria)**	**Subtopic (n criteria)**	**Dimensions to rate**
**Specific**	**A. Content issues (130)**	Information on CRC and CRC screening (32)	CRC screening (12)	Reported: yes / no
			**Aetiology and epidemiology of colorectal cancer (German data) (20)**	Correct: yes / no / unclear
				Presentation format: text / number / chart / Table / figure
				Evidence level reported: yes / no / lack of evidence indicated
				Inclusion of quotes / notes
		Information on screening colonoscopy (66)	Colonoscopy preparation (7)	Reported: yes / no
			Colonoscopy sedation (4)	Correct: yes / no / unclear
			Procedure (13)	Presentation format: text / number / chart / Table / figure
			Test characteristics (7)	
			Conduct in response to test results (3)	Evidence level reported: yes / no / lack of evidence indicated
			**Benefit (disease-specific incidence and total mortality) (9)**	
				Inclusion of quotes / notes
			**Risks and adverse effects including overdiagnosis (23)**	
		Information on FOBT (32)	Procedure (9)	Reported: yes / no
			Test characteristics (8)	Correct: yes / no / unclear
			Conduct in response to test results (3)	Presentation format: text / number / chart / Table / figure
			Benefit (disease-specific incidence and total mortality) (9)	
				Evidence level reported: yes / no / indication of lack of evidence
			Risks and adverse effects including overdiagnosis (3)	
				Inclusion of quotes / notes
**Generic**	**B. Formal issues (33)**	Formal issues (33)	Author and stakeholders involved (14)	Reported: yes / no
			Editorial independence (6)	Inclusion of quotes / notes
			Sources and currentness of data (8)	
			Aim and target group (5)	
	**C. Presentation & understandability (59)**	Readability / comprehensibility (29)	Language (18)	Present: yes / mostly yes / mostly no / no / not applicable
			**Sentences (4)**	
			Content structure (3)	Inclusion of quotes / notes
			**Numerical data (4)**	
		Layout (30)	Structure (11)	Present: yes / mostly yes / mostly no / no / not applicable
			Writing/font (6)	
			**Visual elements (9)**	Inclusion of quotes / notes
			Design (4)	
	**D. Neutrality & balance (7)**	Neutrality and balance (7)	Calls for participation (1)	Present: yes / no / unclear
			Fear / downplay (4)	Inclusion of quotes / notes
			Uneven presentation of procedures (2)	

The bold subtopics are presented in detail in Tables 2–5. The criteria of Domain D are listed in the text.

*One comprehensive criterion on the correctness of the information is not shown.

FOBT: fecal occult blood test.

**Table 2:** Dimensions of the list of criteria (excerpt)

**Risks and adverse effects of screening colonoscopy**					
**Criterion**	**Reported?**	**Correct?**	**Presentation?**	**Evidence level reported?**	**Quotes / notes**
Overall risk of adverse effects of screening colonoscopy is indicated	□ yes	□ yes	□ text	□ yes	
	□ no	□ no	□ number	□ no	
		□ unclear	□ chart	□ lack of evidence indicated	
			□ table		
			□ image		
Risk of pain is indicated	□ yes	□ yes	□ text	□ yes	
	□ no	□ no	□ number	□ no	
		□ unclear	□ chart	□ lack of evidence indicated	
			□ table		
			□ image		
Risk of cardiovascular symptoms is indicated	□ yes	□ yes	□ text	□ yes	
	□ no	□ no	□ number	□ no	
		□ unclear	□ chart	□ lack of evidence indicated	
			□ table		
			□ image		

The preliminary list of criteria was modified in response to the experts’ reviews, mainly by including additional criteria (e.g. inability to drive after sedation, further risks in the preparation phase of colonoscopy, possibility of being unable to work on the day of examination, and the need to sign a consent form and give a blood sample before the examination).

### Final list of criteria

The final list of criteria contains 230 criteria (Table 1). Most of the single criteria are rated multi-dimensionally: reporting: yes/no; correctness: yes/no/unclear; presentation: text, numbers, diagrams, tables and/or images; level of evidence: yes, no, lack of evidence indicated (Table 2). To enhance the rating transparency of each criterion, space for free text is provided for verbatim quotes or reported numbers, to document whether a number was presented as a natural frequency [42,43], and to specify whether a denominator was included, etc.

Elements of the four domains are explained in detail below, including assessment examples, where appropriate.

### Domain A: (CRC-specific) content issues

Domain A includes three categories (see Table 1). The subtopic “Information on the etiology and epidemiology of CRC” of the category “Information on CRC and CRC screening” is presented in Table 3 to elucidate the procedure for detection of epidemiological frequencies. It becomes clear that not all criteria have to be met for information material to qualify as being of high quality. Examples of how information in flyers and brochures of this category were assessed are shown below.

**Table 3:** Criteria for the aetiology and epidemiology of CRC (n = 20) (Domain A, Category: Information on CRC and CRC screening)


1	Meaning of premalignant conditions like polyps is stated.
2	Frequency of polyps/adenomas is stated.
3	Risk factors are stated.
4	Protective measures are stated.
5	Incidence is stated.
6	Sex-specific incidence is stated.
7	Age-specific incidence (age-stratified incidence) is stated.
8	Mortality is stated.
9	Sex-specific mortality is stated.
10	Age-specific mortality is stated.
11	Residual lifetime disease risk is stated.
12	Residual lifetime risk of death is stated.
13	Age-specific disease risk within a given time interval is stated.
14	Age-specific mortality risk within a given time interval is stated.
15	The disease risk compared to other cancer disease risks is stated.
16	The disease risk compared to other risks is stated.
17	The mortality risk compared to other cancer mortality risks is stated.
18	The mortality risk compared to other risks of death is stated.
19	The natural course of CRC is stated.
20	Incidence and mortality are not stated in one sentence.

Example 1: “*CRC is the second most common type of cancer in both men and women.*”

This statement would be rated as criterion 15 (Table 3):

–Reported? “Yes”

–Correct? “Yes”

–How presented: “Text” (not “Number”)

–Evidence level reported? “Not applicable”

–Quotes: Citation

Example 2: “*22,000 people die each year from CRC.*”

This statement would be rated as criterion 8 (Table 3):

–Reported? “Yes”

–Correct? “No” (number is too low for Germany)

–How presented: “Number”

–Evidence level reported? “No”

–Quotes: “Denominator is lacking, outdated number”

Both examples show the importance of having a manual that provides the correct answers and numbers and, in the second case that defines what extent of deviation from the actual number is acceptable as “correct”. Therefore, the manual is a core part of the list of criteria.

The categories of the two screening procedures, fecal occult blood test and colonoscopy, are constructed similarly. They begin with information on the procedure itself and are supplemented by further criteria on colonoscopy preparation and sedation. Both procedures incorporate criteria on test characteristics (such as sensitivity, specificity, predictive value), on conduct in response to test results and, most importantly, on benefit and risks, including overdiagnosis.

Table 4 shows the criteria on the subtopics of benefits and risks of screening colonoscopy. Benefits include three relevant outcomes: CRC incidence, CRC mortality and all-cause mortality. Each outcome is divided into absolute and relative risk reduction and the number needed to screen. Risk criteria for screening colonoscopy are divided into risks during colonoscopy preparation (including colon cleansing), risks related to adverse effects of sedative drugs, risks of the procedure itself, and risks of overdiagnosis. The subject of overdiagnosis is included because it is known to occur in cancer screening to a varying extent depending on the type of cancer [44-46]. Nevertheless, the extent of overdiagnosis or overtreatment of harmless polyps that would never turn into cancer in colorectal cancer screening is unknown and may be low as there are strong hints that colonoscopy will decrease CRC incidence like it is already shown for flexible sigmoidoscopy-based screening [47]. The rating procedure for benefits and risks is illustrated below.

**Table 4:** Criteria on benefits (n = 9) and risks (n = 23) of screening colonoscopy (Domain A, Category: Information on screening colonoscopy)

**Benefits of screening colonoscopy**	
Outcome: CRC incidence	
1	Absolute risk reduction is stated.
2	Relative risk reduction is stated.
3	Number needed to screen is stated.
Outcome: CRC mortality	
4	Absolute risk reduction is stated.
5	Relative risk reduction is stated.
6	Number needed to screen is stated.
Outcome: All cause mortality	
7	Absolute risk reduction is stated.
8	Relative risk reduction is stated.
9	Number needed to screen is stated.
**Risks of screening colonoscopy**	
Preparation	
1	Common risk of side effects is stated.
2	Risk of cardiovascular symptoms is stated.
3	Risk of nausea is stated.
4	Risk of allergies is stated.
5	Risk of cramps is stated.
6	Risk of pain is stated.
Sedation	
7	Common risk of side effects is stated.
8	Risk of respiratory distress/failure is stated.
9	Risk of cardiovascular symptoms is stated.
10	Risk of Nausea is stated.
Procedure itself	
11	Common risk of side effects is stated.
12	Number needed to harm is stated.
13	Risk of pain is stated.
14	Risk of cardiovascular symptoms is stated.
15	Risk of Nausea is stated.
16	Risk of bleeding is stated.
17	Risk of infection is stated.
18	Risk of perforation is stated.
19	Risk of mortality is stated.
Overdiagnosis	
20	Risk of overdiagnosis/overtreatment is stated.
21	Frequency of overdiagnosis is stated.
23	Consequences of overdiagnosis are stated.

Example 3: “*According to experts, more than three-quarters of CRC patients could be saved by early screening colonoscopy.*”

This statement would be rated as criterion 5, CRC mortality (Table 4: Benefits):

–Reported? “Yes”

Correct? “Yes”

–Presented as: “Number”

–Evidence level reported? “Yes”

–Quotes: “No natural frequency, denominator is lacking, no absolute risk reduction is given, evidence from level 3 (case-control) studies is falsely presented as experts’ evidence”.

Example 4: “*80% of all CRCs can be prevented by screening colonoscopy.*”

This statement would be rated as criterion 2, CRC incidence (Table 4: Benefits):

–Reported? “Yes”

–Correct? “Yes”

–Presented as: “Number”

–Evidence level reported? “No”

–Quotes: “No natural frequency, denominator is lacking, no absolute risk reduction is given, evidence from level 3 (case–control) studies

Example 5: “… *is a harmless drug preparation*”

This statement would be rated as criterion 1 (Table 4: Risks):

–Reported? “Yes”

Correct? “No”

–Presented as: “Text”

–Evidence level reported? “No”

–Quotes: Citation

Example 6: “*… no pain*”

This statement would be rated as criterion 12 (Table 4: Risks):

–Reported? “Yes”

–Correct? “No”

–Presented as “Text”

–Evidence level reported? “No”

–Quotes: Citation

### Domain B: formal issues (generic)

This domain assesses the formal characteristics of information materials, including information on the authors and editors, possible conflicts of interest, publication dates, aims and target groups. Due to the nature of this meta-information, only the presence or absence of these criteria is rated and not their correctness. As criteria in this domain are very are widely used, we do without describing them in detail.

### Domain C: presentation and understandability (generic)

“Understandability (readability/comprehensibility)” assesses the language, sentences, content structure, and numbers of information materials, whereas “presentation (layout)” concerns the structure, font, visual elements and design of the materials (see Table 1). These criteria (e.g., “Sentences are of appropriate length”) require more detailed rating, such as that achieved by four response categories. Therefore, all criteria in this domain were rated on a four-point-scale (yes / mostly yes / mostly no / no). Furthermore, it makes no sense to rate the correctness of these criteria. For most of the assessments in this domain, it is essential to aggregate information: For example, when assessing the length of a sentence, the assessor must search the entire health information material for sentences that are too long. To ensure an unambiguous assessment, the manual should provide a definition of what is “too long” and what proportions of run-on long sentences should lead to which specific ratings. Table 5 provides a detailed list of criteria for sentences, numbers and visual elements followed by a rating example for this category.

**Table 5:** Criteria for sentences (n = 4), numerical data (n = 4) and visual elements (n = 9) (Domain C, sub topics from both categories)

**Understandability**		**Presentation**	
	**Sentences**		**Visual elements**
1	There is one message per sentence.	1	Visual elements are included.
2	Sentences are of appropriate length.	2	Drawings are used instead of photos.
3	Complex sentences are avoided.	3	Visual elements are explained in the text.
4	Identical repetitions are avoided.	4	The explanatory text is near the visual element.
	**Numerical data**	5	The visual element is not surrounded by text.
1	Natural frequencies are used.	6	Visual elements are clearly labeled.
2	Reference parameters are given.	7	Biased scaling is avoided.
3	Same denominators are used.	8	Important spots of the visual element are marked by arrows, circles etc.
4	Loss and gain framing is balanced.	9	Visual elements include a legend.

Example 7: *“every year 70,000 persons are newly diagnosed with colorectal cancer.”*

Provided that this is the only number given in the health information, this statement would be rated as follows:

–Numerical data criterion 1: Natural frequencies are used? “Yes”

–Criterion 2: Reference parameters are given? “No”

–Criterion 3: Same denominators are used? “No”

–Criterion 4: Loss and gain framing is balanced? “No”

Usually, several numbers are stated in a text. In that case, an aggregated assessment is required.

### Domain D: neutrality and balance (generic)

The last domain comprises seven criteria for assessment of neutral and balanced presentation:

1 “Is free of persuasive language”

2 “Is free of scare language”

3 “Is free of scary pictures or graphs”

4 “Is free of fear appeals”

5 “Is free of downplay or minimization”

6 “Is free of one-sided presentation of benefits without risks”

7 “Is free of unbalanced presentation of screening procedures”

The first five criteria are rated “no”, if any persuasive, scary or down-playing language is used to increase participation in screening. We initially defined these criteria as “Does not contain….”, but this phrase was abandoned because the possible double-negative reply might be confusing. The last two criteria combine benefits and risks and presentation of the procedures. To handle this aggregate information, careful operationalization within the manual is needed. Rating examples for this category are given below.

Example 8: “*… should participate in bowel cancer screening.*”

This statement would be rated as criterion 1:

Met? “Yes”.

Example 9: “*… is a wicked disease*”

This statement would be rated as criterion 2:

Met? “Yes”

### Applications / practicability

For trained reviewers, the assessment takes about 15–30 minutes for flyers and 15–45 minutes for brochures. Documentation of the corresponding citations took up much of the time. Although this approach may be time-consuming, it may hasten consensus and, most importantly, ensures the transparency of quality assessment.

Inter-rater reliability was not evaluated because the final assessment was achieved by consensus in each case. Discrepant findings were mainly caused by overlooked aspects. Consensus usually took 5 to 15 minutes. Finally, data entry is very time-consuming due to the citations.

### Possible methods of presenting rating results

**Figure 2:**
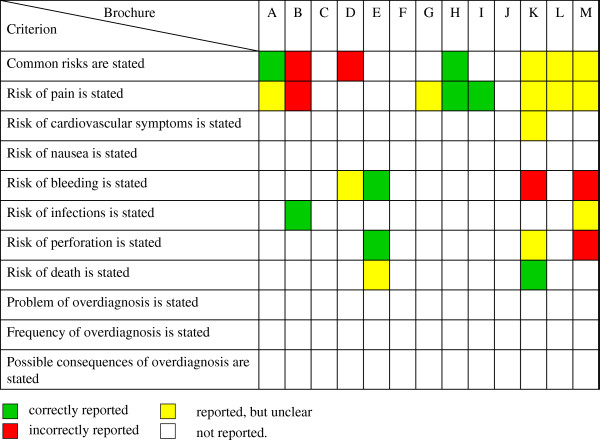
Presentation of exemplary rating results of risks and adverse effects of screening colonoscopy in 13 brochures (11 criteria).

Quantitative analyses can be made on the level of criteria and their dimensions. Until now, none were performed on the level of subtopics, categories or domains. A method to qualitatively sum up the single-criteria results is also lacking. To obtain an overview, the combined results of the two dimensions “Reported?” and “Correct?” can be visualized by means of a traffic light system, using green (correctly reported), yellow (reported but unclear), red (incorrectly reported), and white (not reported) marks. Figure 2 gives an example from the category “Risks and adverse effects of screening colonoscopy” as presented in brochures. This (traffic light) presentation provides a comprehensive overview of results for each criterion (rows), each type of education material (columns), and differences between materials as in benchmarking procedures. It clearly shows that information on risks is rather limited and sometimes false, and that three brochures contain no information on risks.

## Discussion

High-quality patient information materials may help consumers make informed decisions for or against participation in CRC screening. For identification of appropriate information materials, we compiled a list of criteria via qualitative aggregation based on systematically identified recommendations and tools, and validated the list of criteria by a review process. This instrument is designed for use by persons with expertise in cancer screening. It explicitly is not a checklist for consumers to check the quality of health information. The final manual-based list of criteria contains 230 criteria in four domains. The criteria are rated on multiple dimensions (e.g., presence *and* correctness of information), if applicable. Free text entries (mainly verbatim quotes) were allowed to ensure rating transparency.

One main limitation of the list of criteria is the lack of a summary assessment. It may be tempting to use a numerical scoring system for simplicity, but there is no empirical evidence to support this. Consequently, as there is no justification for a scoring system that applies an arbitrary (one point per criterion, etc.) or explicit weighting system, we rejected the use of a numerical scoring system or scale. Scales for quantitative assessment of study quality were very popular until empirical evidence [48] and theoretical considerations [49] indicated that scales provide invalid results. Because of the lack of a summary assessment method, analyses with the proposed list of criteria are restricted to the level of single criteria. This is inconvenient due to the large number of criteria to be assessed. To give an overview of the results, we used a traffic light (status indicator) system. This system can indicate two dimensions (e.g., presence and correctness of information) simultaneously, and it provides detailed information on single and overall categories. Such a comparative overview is particularly useful for benchmarking purposes. The future aim is to develop a qualitative summary assessment based on ratings on the category level. A Delphi consensus process might be used to explore the importance of each criterion, as was done in International Patient Decision Aids Standard Instrument (IPDASi) development [14].

The list of criteria represents the maximum content of information material. Not all of the criteria are essential for high-quality information. The comprehensiveness and depth of information materials vary depending on the targets and target groups. Thus, it would be reasonable to differentially define essential criteria for short information materials like flyers and for more detailed materials like brochures. It would also be reasonable to select these mandatory, material-specific criteria in a Delphi procedure including experts and consumers. Obviously, expert and consumer opinion is needed to explore the importance of each criterion for further summary assessments and to develop specific assessment lists of criteria for short and more detailed information materials. This input could be used in further research to revise the list of criteria.

The rationale behind providing EBHI on cancer screening is to enable consumers to make informed choices for or against cancer screening. The proposed list of criteria examines whether health information materials meet EBHI standards. It cannot directly assess whether the information is suitable to support informed decision-making. There is evidence from two randomized controlled trials that decision aids on CRC screening via FOBT [8] and FOBT/colonoscopy [7] may effectively support informed choice. However, both studies compared an interactive decision booklet with an accompanying DVD [8] or interactive internet module [7] (intervention groups) against a standard governmental booklet (control group). The effect may have been mediated by the interactive components resulting in more intense study of the materials. The proposed list of criteria does not assess interactive components. Ultimately, if information materials are found to meet EBHI standards according to our list of criteria, it cannot be concluded that these materials promote informed choice. Such a claim would have to be verified in further studies.

The ethical goal of EBHI to enable as many of the target population to make an informed decision whether or not to participate in CRC screening [3] may be conflicting with the aim of achieving a high uptake [50]. There is inconclusive evidence on detailed information material, it may have a positive or no effect on participation [7,10] or may even increase non-attendance [8]. Non-attendance based on an informed choice has to be accepted, while non-attendance arising from the EBHI itself and not from an informed choice is not desirable. EBHI especially may deter socioeconomically disadvantaged people and those with low health literacy from participating in screening [51] resulting in higher health inequalities. Further research is needed to explore tailored communication strategies for deprived target groups focusing on increasing knowledge and understanding to promote an informed choice-making.

To our knowledge, the proposed list of criteria is the first assessment tool designed to rate the correctness of consumer education materials on CRC screening. Many existing tools use structural quality as a surrogate for content quality, which might not always be correct. For example, it was shown that website origin does not predict content quality: the quality of university websites was not better than that of commercial websites [52]. Website certification programs like the HON (Health on the Net) code [53] and MedCERTAIN (MedPICS Certification and Rating of Trustworthy Health Information on the Net) were also established to ensure the provision of reliable information. The HON code requires health information website owners to abide by eight principles: to indicate the authors’ qualifications, information sources, funding sources and advertising policy and to maintain confidentiality, etc. However, there are concerns that these criteria might not be sufficient to identify trustworthy information. For example, the HON label failed to predict the good content quality of mental health-related websites in some cases [52]. In contrast, the DISCERN score was shown to be a content quality indicator of relatively high specificity [52,54]. Other analyses of web-based information on depression found that content quality correlated with the DISCERN score and HON label [55]. However, the usual tools for the assessment of content quality do not check the correctness of information and might even rate false information as being of good quality.

As the proposed manual provides the correct answers to the criteria queries, it is an essential part of the rating method. Ideally, many CRC-specific content criteria should be explored by systematic reviews, especially if related to benefits and risks. This was not feasible in this project. Our research, which was mainly restricted to selectively searched evidence from S3 guidelines, systematic reviews and HTA reports, was still very time-consuming. It will be challenging to incorporate the latest evidence in the finished manual, as knowledge changes over time. It is also unclear how often the manual should be updated. Other problems can arise from different interpretations of the evidence. In breast cancer screening, for example, experts disagree on the actual numbers characterizing the benefits and risks in a British leaflet [56]. The National Cancer Institute (USA) took an interesting approach to providing key information, namely, by posting a one-page factsheet on lung cancer screening for doctors and patients providing numbers on benefits and risks derived from a randomized controlled trial [57]. In Europe, aggregated uniform evidence-based factsheets on screening procedures would complement the existing comprehensive guidelines [3] and would offer a thorough base of knowledge for different players who provide information on cancer screening.

## Conclusions

The range of the proposed evaluation concept based on a list of 230 criteria and answer manual goes beyond that of previous instruments for quality assessment of health information in that it considers not only the presence, but also the correctness of health information. However, this comprehensive list of criteria is not intended as a final quality assessment tool, but rather as a first step toward thorough evaluation of specific information materials for adherence to EBHI standards made by persons with professional expertise in cancer screening. It may also be used to revise existing leaflets or to develop health information materials on colorectal cancer screening. Furthermore, the proposed list of criteria can be transferred to other cancer screening procedures after suitable modification of the procedure-specific criteria.

## Abbreviations

CRC: Colorectal cancer; EBHI: Evidence-based health information.

## Competing interests

The authors declare that they have no competing interests.

## Authors’ contributions

BB performed the literature search. MD, BB, GS and IK selected the literature, extracted the criteria, developed the list of criteria, and tested the final list of criteria. UW, MLD, JT, and EMB critically revised the list of criteria and read and approved the final manuscript, especially UW and MLD. MD and BB drafted the manuscript. All authors participated in interpretation of the results and critically revised subsequent versions of the paper for important intellectual content. All authors read and approved the final manuscript.

## Pre-publication history

The pre-publication history for this paper can be accessed here:

http://www.biomedcentral.com/1471-2458/13/843/prepub

## Supplementary Material

Additional file 1 Search strategy in electronic databases. Click here for file
