# Supplementary material for: Development of a comprehensive list of criteria for evaluating consumer education materials on colorectal cancer screening
Source: BMC Public Health. 2013 Sep 13;13:843. doi: 10.1186/1471-2458-13-843 (PMC3848725; doi:10.1186/1471-2458-13-843)
Supplement: Additional file 1 — Search strategy in electronic databases. [file 1471-2458-13-843-S1.doc]

**Additional file 1**

## Table 1: Search strategy in electronic databases (8/2010)

| 1 | ME00 BA00 DAHTA AR96 EA08 EM00 GA03 GM03 KR03 KP05 IS00 TVPP TV01 |
| --- | --- |
| 2 | TI=criteria OR (CT D "criteria" OR UT="criteria" OR IT="criteria" OR SH="criteria") |
| 3 | TI=quality OR (CT D "quality" OR UT="quality" OR IT="quality" OR SH="quality") |
| 4 | TI=assessment of quality OR (CT D "assessment of quality" OR UT="assessment of quality" OR IT="assessment of quality" OR SH="assessment of quality") |
| 5 | TI=assessing the quality OR (CT D "assessing the quality" OR UT="assessing the quality" OR IT="assessing the quality" OR SH="assessing the quality") |
| 6 | TI=checklist OR (CT D "checklist" OR UT="checklist" OR IT="checklist" OR SH="checklist") |
| 7 | ((TI=appraisal OR (CT D "appraisal" OR UT="appraisal" OR IT="appraisal" OR SH="appraisal")) OR TI=quality appraisal ) OR (CT D "quality appraisal" OR UT="quality appraisal" OR IT="quality appraisal" OR SH="quality appraisal") |
| 8 | 2 OR 3 OR 4 OR 5 OR 6 OR 7 |
| 9 | ((TI=patient decision aid OR (CT D "patient decision aid" OR UT="patient decision aid" OR IT="patient decision aid" OR SH="patient decision aid")) OR TI=decision aid ) OR (CT D "decision aid" OR UT="decision aid" OR IT="decision aid" OR SH="decision aid") |
| 10 | ((TI=Informed choice OR (CT D "Informed choice" OR UT="Informed choice" OR IT="Informed choice" OR SH="Informed choice")) OR TI=informed decision ) OR (CT D "informed decision" OR UT="informed decision" OR IT="informed decision" OR SH="informed decision") |
| 11 | ((TI=patient information OR (CT D "patient information" OR UT="patient information" OR IT="patient information" OR SH="patient information")) OR TI=health information ) OR (CT D "health information" OR UT="health information" OR IT="health information" |
| 12 | (((TI=shared decision OR (CT D "shared decision" OR UT="shared decision" OR IT="shared decision" OR SH="shared decision")) OR TI=decision making ) OR (CTG D "decision making" OR UTG="decision making" OR ITG="decision making" OR SHG="decision making")) |
| 13 | TI=Risk communication OR (CT D "Risk communication" OR UT="Risk communication" OR IT="Risk communication" OR SH="Risk communication") |
| 14 | ((TI=consumer health information OR (CT D "consumer health information" OR UT="consumer health information" OR IT="consumer health information" OR SH="consumer health information")) OR FT=Evidence-based patient information ) OR (CT D "Evidence-based patient information") |
| 15 | 9 OR 10 OR 11 OR 12 OR 13 OR 14 |
| 16 | ((TI=information brochure# OR (CT D "information brochures" OR UT="information brochures" OR IT="information brochures" OR SH="information brochures")) OR (CT D "pamphlets" OR UT="pamphlets" OR IT="pamphlets" OR SH="pamphlets")) OR (CT D "consumer information") |
| 17 | 15 OR 16 |
| 18 | 8 AND 17 |
| 19 | 18 AND PY=2000 to 2010 |
| 20 | 19 AND LA=(ENGLISH; GERMAN) |
| 21 | 20 AND (pps=Mensch OR pps=Krebserkrankungen) |
| 22 | (((CTG D "quality of life" OR UTG="quality of life" OR ITG="quality of life" OR SHG="quality of life") OR (CT D "quality of life" OR UT="quality of life" OR IT="quality of life" OR SH="quality of life")) OR TI=quality of life ) OR FT=quality of life |
| 23 | 21 NOT 22 |
| 24 | ((CT D "pregnant" OR UT="pregnant" OR IT="pregnant" OR SH="pregnant") OR (CT D "pregnancy" OR UT="pregnancy" OR IT="pregnancy" OR SH="pregnancy")) OR FT=pregnan* |
| 25 | 23 NOT 24 |
| 26 | ((CT D "genetic" OR UT="genetic" OR IT="genetic" OR SH="genetic") OR (CT D "genetics" OR UT="genetics" OR IT="genetics" OR SH="genetics")) OR FT=genetic* |
| 27 | 25 NOT 26 |

## Table 2: Search strategy in the Internet with “google” (8/2010)

| Search terms in English |
| --- |
| (criteria OR quality OR checklist OR appraisal) AND "patient information" |
| (criteria OR quality OR checklist OR appraisal) AND "health information" |
|  |
| (criteria OR quality OR checklist OR appraisal) AND "Informed choice" AND (patient OR patients OR patienten OR health OR gesundheit ) |
| (criteria OR quality OR checklist OR appraisal) AND "Informed decision" AND (patient OR patients OR patienten OR health OR gesundheit) |
| (criteria OR quality OR checklist OR appraisal) AND "shared decision" AND (patient OR patients OR patienten OR health OR gesundheit) |
| (criteria OR quality OR checklist OR appraisal) AND "decision making" AND (patient OR patients OR patienten OR health OR gesundheit) |
| (criteria OR quality OR checklist OR appraisal) AND "decision aid" AND (patient OR patients OR patienten OR health OR gesundheit) |
| (criteria OR quality OR checklist OR appraisal) AND ("risk communication") AND (patient OR patients OR patienten OR health OR gesundheit) |
|  |
| (criteria OR quality OR checklist OR appraisal) AND "pamphlets" AND (patient OR patients OR patienten OR health OR gesundheit) |
| (criteria OR quality OR checklist OR appraisal) AND "brochures" AND (patient OR patients OR patienten OR health OR gesundheit) |
|  |
| Search terms in German |
| (Qualitätskriterien OR Kriterien OR Qualität OR Checkliste OR bewertung) AND "Patientenbroschüren" |
| (Qualitätskriterien OR Kriterien OR Qualität OR Checkliste OR bewertung) AND "Patienteninformation" |
| (Qualitätskriterien OR Kriterien OR Qualität OR Checkliste OR bewertung) AND "Patienteninformationen" |
| (Qualitätskriterien OR Kriterien OR Qualität OR Checkliste OR bewertung) AND "gesundheitsinformation" |
| (Qualitätskriterien OR Kriterien OR Qualität OR Checkliste OR bewertung) AND "gesundheitsinformationen" |
|  |
| (Qualitätskriterien OR Kriterien OR Qualität OR Checkliste OR bewertung) AND "information" AND (Gesundheit OR Patienten) |
| (Qualitätskriterien OR Kriterien OR Qualität OR Checkliste OR bewertung) AND "informationen" AND (Gesundheit OR Patienten) |
| (Qualitätskriterien OR Kriterien OR Qualität OR Checkliste OR bewertung) AND "informationsmaterial" AND (Gesundheit OR Patienten) |
| (Qualitätskriterien OR Kriterien OR Qualität OR Checkliste OR bewertung) AND "informationsmaterialien" AND (Gesundheit OR Patienten) |
